# Does Cataract Surgery Influence Iris-Based Biometric Authentication?

**DOI:** 10.22336/rjo.2025.30

**Published:** 2025

**Authors:** Sonali Vinay Kumar, Vinay Kumar, Natasha Vinay Kumar, Alok Sati, Sanjay Kumar Mishra

**Affiliations:** 1 Command Hospital Eastern Command, Kolkata, West Bengal, India; 2 JIS School of Medical Science and Research, Howrah, West Bengal, India; 3 Department of Medicine, Sri Devaraj Urs Medical College, Kolar, Karnataka, India; 4 471 Field Hospital, Arunachal Pradesh, India; 5 Army Hospital Research and Referral, Delhi, India

**Keywords:** iris recognition, cataract surgery, iris camera, biometric, uneventful surgery, Hamming distance, UIDAI = Unique Identification Authority of India, HD = Hamming distance

## Abstract

**Purpose:**

This study investigates the impact of cataract surgery on the reliability and accuracy of iris recognition systems used for biometric identification.

**Methods:**

We carried out a prospective observational study on patients undergoing cataract surgery via phacoemulsification. The study comprised 100 participants who underwent cataract surgery. We recorded preoperative iris scans using a standard biometric device (iris camera) and rescanned the same patients after surgery. Matching scores before and after surgery were analyzed to detect discrepancies. We examined the Hamming distance to evaluate changes in the iris pattern.

**Results:**

The mean age of the study population was 64 ± 6.2 years (range, 52-74 years). The study population comprised 62 males and 38 females. Out of the total cases, 72 involved the right eye and 28 involved the left eye. The majority of patients presented within the current study presented with visual acuity between 6/18 and 3/60. We performed phacoemulsification and implanted foldable intraocular lenses in all cases. The study found no notable changes in iris pattern matching accuracy following uncomplicated cataract surgery. Postoperative images were matched with preoperative images in 95 patients, demonstrating that the surgery did not affect biometric reliability. The mean Hamming distance before and after surgery remained within the acceptable threshold for authentication, with 99% of cases successfully matched.

**Discussion:**

The study supports the robustness of iris-based biometric systems under routine ophthalmic surgical conditions. This system’s resilience to variations caused by cataract surgery demonstrates its robustness in practical sessions.

**Conclusion:**

Cataract surgery, when performed without complications, does not impair the accuracy of iris-based biometric identification. These findings underscore the feasibility of using iris recognition systems in healthcare and security, even among patients undergoing ocular procedures.

## Introduction

Iris recognition and scanning are among the most reliable biometric methods for personal identification. This technology is currently utilized in various applications, including Aadhaar authentication by the Unique Identification Authority of India (UIDAI), banking systems, security services, and international border security [1,2].

The human iris has a complex structure with distinct features, including furrows, freckles, and crypts. These features are unique and different due to the individual variations that arise during embryonic development. The iris remains highly stable throughout a person’s lifetime and is well-suited for non-invasive identification due to its external visibility [3,4]. Governmental and corporate bodies worldwide are adopting biometric authentication systems that use fingerprint, facial, and iris recognition, with the latter being crucial for de-duplication in large-scale national and international databases. It is therefore essential to understand the impact of standard procedures and medications on iris recognition performance [5,6].

Various intraocular diseases and surgical procedures can alter the iris architecture, potentially compromising iris recognition systems and resulting in severe security and financial consequences. Worldwide, ophthalmologists most commonly perform cataract surgery among all ocular procedures. In India, approximately 8 million cataract surgeries are performed annually. This procedure may result in deformation of the iris during surgery and a shift in the iris position away from the cornea due to the replacement of the lens with an intraocular lens. Therefore, understanding the impact of cataract surgery on iris biometric recognition is essential. This detailed analysis of the effects of cataract surgery on iris recognition aims to enhance our understanding of the limitations and strengths of generic iris-based biometric systems.

There is limited data from India and globally regarding changes in iris patterns following cataract surgery. Therefore, the current study was undertaken to evaluate the impact of cataract surgery on iris recognition systems and iris pattern stability.

## Methods

### Design

This prospective cohort study was conducted in the Department of Ophthalmology at a tertiary care center between January 2023 and June 2023.

This study adhered to the principles outlined in the Declaration of Helsinki. We conducted the study for six months and followed all patients for an additional six months.

The research team enrolled 100 patients scheduled for cataract surgery. We excluded patients with any other ocular disease, iris atrophy, or a history of intraocular surgery. The exclusion criteria included corneal pathology, pre-existing iris abnormalities, intra-operative complications such as iris damage, inability to maintain stillness during assessment (head tremor, dementia), inability to give informed consent, and refusal to participate.

**Fig. 1:**
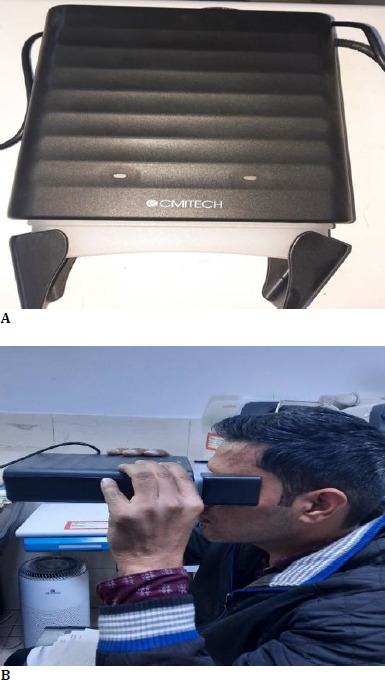
**A**. Clinical photograph showing an iris scan used in the study; **B**. Clinical photograph showing a patient having his eyes imaged with an iris camera

The examiner asked each patient to position themselves correctly in front of the iris camera and to achieve maximum eye opening. We conducted the procedure in the same room, maintaining constant illumination (horizontal and vertical 70 lux) and a fixed distance from the equipment to ensure ideal laboratory conditions. We enrolled participants using iris camera scans and processed the captured images through software for analysis and recognition. All iris images were captured using a BMT-20 (CMI TECH) iris camera (**Fig. 1**).

After enrolment, we performed three trials and recorded the Hamming distance and focus data. After enrolling the participants, we performed post-surgical identification trials for one week, one month, and six months. The surgeon performed cataract surgery using the phacoemulsification technique and implanted an intraocular lens in all cases. Given the favorable experimental setup, three trials were not strictly required. However, since elderly patients may occasionally sneeze or cough during the procedure, we performed three trials and selected the most accurate one for analysis. We incorporated pupillary size adjustments into the algorithm. We avoided pharmacological dilatation, and the measured changes in pupil size remained below 1.5 mm. Significant iris changes typically occur in the early postoperative period due to surgical manipulation. Acute healing, along with chronic tissue retraction, is normally complete within one month; therefore, the testing period was selected accordingly. The mean preoperative Hamming distance (HD) was compared with the mean postoperative Hamming distance. The Hamming distance threshold was set at 0.33, aligning with the standard used in many indoor public settings. We assessed the variation in Hamming distance for each subject by comparing the preoperative measurement with the lowest postoperative reading. We opted for the lowest value, considering it to represent the clearest and most precise image for biometric coding. The primary endpoint of the study was whether the postoperative iris scan matched the enrolment scan for each individual. The research team used SPSS software to assess the study endpoint and applied ANOVA and Student’s t-test to evaluate statistical differences.

The matching process begins when a user enrolls in the biometric system by providing biometric data, which the system converts into a template. Templates are compact data files, known as iris codes, which consist of optimized and filtered images acquired from biometric photos. The biometric system saves these templates for subsequent matching and identification. The user then provides their biometric data again, which the system uses to generate a new template. We compared the verification template with the enrollment template and computed the difference between the corresponding iris codes. This mathematical difference is called the Hamming distance (HD). The Hamming distance, which quantifies the differences between two iris templates, is used as a matching score. This value is assessed against a preset threshold specific to the system or application, resulting in either an identification or a rejection.

## Results

**Fig. 2:**
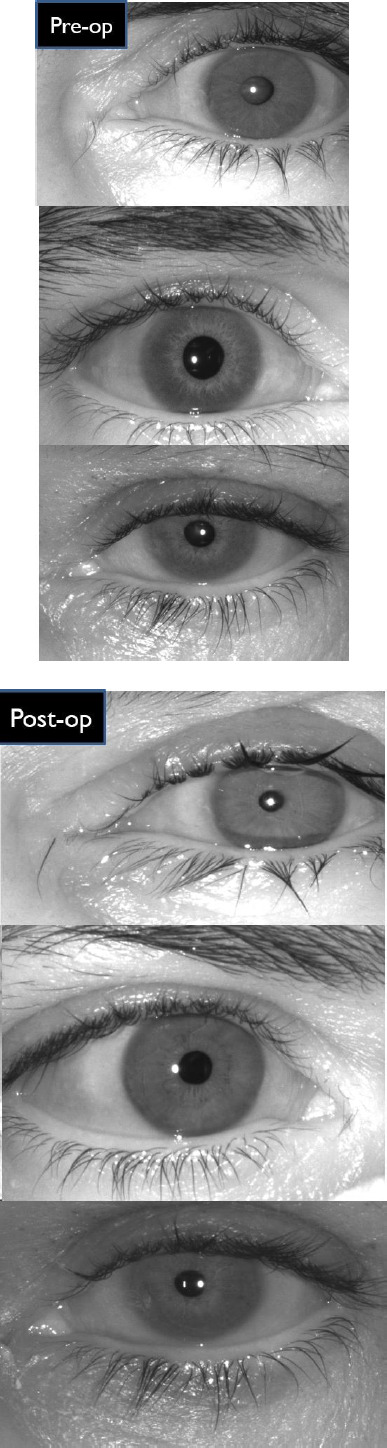
Iris images before and after cataract surgery captured by the iris camera

**Fig. 3:**
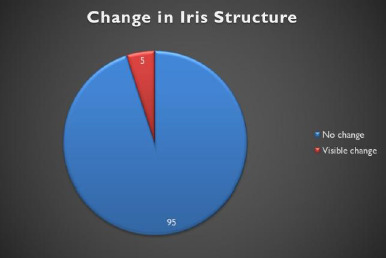
Clinical outcomes in the current study

We enrolled hundreds of cataract patients in this study over the course of one year, based on established inclusion criteria. The mean age of participants was 64 ± 6.2 years (range: 52 to 74 years) in this study. The current study included 62 male and 38 female participants. Examiners noted right eye involvement in 72 patients and left eye involvement in 28 patients. The majority of patients in the current study presented with visual acuity ranging from 6/18 to 3/60. We performed phacoemulsification and implanted a foldable intraocular lens (IOL) in all patients. The comparison of pre- and post-cataract surgery images yielded good results. We observed no visible alterations in iris structure in 95 postoperative cases; however, five patients showed changes that affected the iris recognition system. We found that in 95 cases, cataract surgery caused temporary deformation, but the iris structure remained largely unaffected (**Fig. 2, 3**).

**Fig. 4:**
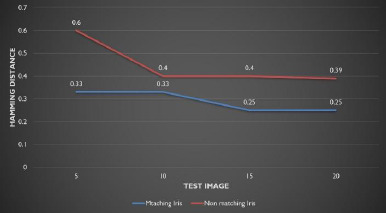
Hamming distance (HD) for matching and nearest non-matching iris after cataract surgery

The intraocular lens implant causes multiple reflections of the infrared light source, leading to additional specular reflections within the pupil. Such reflections could be a potential cause for concern for light-position-based pupil-finding algorithms. The localization process used in this study finds the darkest region of the significant area nearest to the image center and is unaffected. We calculated the Hamming distance (HD) for matching and nearest non-matching undilated irises after cataract surgery. A clear separation between matches and non-matches indicates perfect verification and identification, with both false acceptance and false rejection rates at zero (**Fig. 4**).

All images captured met the criteria for optimal acquisition, with focus values exceeding 95% on a 100% scale (acceptable above 70%) and image quality scores above 1200 on a scale of up to 1600 (acceptable above 600). All cases demonstrated consistent maximum ocular opening both before and after surgery, as confirmed by images captured during iris code generation.

Five patients could not be recognized after the procedure, so their eyes were re-enrolled to create new iris templates. All other patients were successfully recognized despite numerical and clinical changes. The average Hamming distance remained at 0.33 in both the preoperative and postoperative periods for 95 patients. However, in five patients, it increased from 0.33 to 0.44 (**Table 1**).

**Table 1:** Comparison of preoperative and postoperative hamming distance between groups

Group	No of patients	Baseline	Post op. day-1	One week	1 month	6 months	p-value
Matching Iris	95	0.33±0.02	0.34 ± 0.02	0.33±0.02	.33±0.02	0.33±.02	p>0.001
Non-matching Iris	5	0.34±.02	0.45±0.02	0.44±0.02	0.44±0.02	0.44±.02	p<0.001

Values expressed as mean ±SD. All measurements are in score. A p-value less than .05 was considered statistically significant.

Although postoperative factors such as transient variations in pupil size or clarity of the ocular surface were considered, their impact on iris recognition was negligible

## Discussion

Iris recognition is one of the most reliable technologies available for person identification. The accuracy of iris recognition technology has made it an increasingly vital tool in biometric identification. The distinctiveness of iris patterns has led to the adoption of iris recognition systems in several large-scale applications. Cataract is one of the leading ophthalmic conditions worldwide, and cataract surgery is routinely performed to restore vision. Cataract surgery has the potential to alter specific ocular parameters. The accurate capture of iris patterns depends on the clarity of the ocular media, making it necessary to determine whether cataract surgery influences iris recognition performance. Post-surgical changes, such as inflammation, pupil size variation, or a shift in light reflection, can potentially impact iris recognition accuracy. This study investigated the accuracy and reliability of the iris recognition system after uneventful cataract surgery performed via phacoemulsification, the most advanced and frequently employed technique.

Initial data from pseudophakic eye evaluations reveal that iris structure remained stable post-surgery in the enrolled subjects. We did not observe any structural changes in the iris, and accurate localization was achieved even with an increased presence of specular reflections in the pupil. Perfect identification was achieved in 95% of the current study population, indicating that the procedure may have little or no effect on an iris-based authentication system. Iris recognition depends on distinct iris patterns, which typically remain structurally and anatomically preserved during cataract surgery. The extracted lens plays no role in creating iris patterns, ensuring that biometric data remains intact after surgery. To validate these results, the study needs to be conducted on a larger population. The findings of the current study were not in agreement with those of Rosenblatt et al., who conducted a survey in South America and concluded that cataract surgery alters iris structure to an extent that iris pattern recognition may no longer be feasible or may increase the probability of false rejections [7]. They recommended re-enrolment of patients who have undergone intraocular procedures in iris biometric systems to create a new template in the database.

Rosenblatt et al. were the first to investigate the impact of cataract surgery on iris pattern recognition [7]. They hypothesized that the failure of iris recognition systems after cataract surgery resulted from changes such as depigmentation, localized iris atrophy, and loss of distinct iris features like Fuchs’ crypts, circular and radial furrows, as well as altered pupil localization. Dhir et al. conducted a study on a limited cohort of 15 individuals and concluded that cataract surgery does not change the iris pattern [8]. According to Preethi et al., cataract surgery leads to structural changes in the iris, and they advised re-enrolling patients for accurate biometric identification [9]. Nigam et al. recently demonstrated that inaccurate segmentation of the iris region in various ophthalmic conditions significantly contributes to the reduced accuracy of advanced iris recognition systems [10]. It is imperative to enhance existing segmentation algorithms to enable precise delineation of the iris region in preoperative and postoperative cataract surgery images, thereby ensuring the reliability of iris recognition systems.

A few additional studies in the literature have examined the effect of cataract surgery on iris patterns.

Seyeddain et al. evaluated the reliability of iris recognition following phacoemulsification cataract surgery and drug-induced pupil dilatation [11]. They found that 94.8% of eyes were successfully recognized postoperatively, while 5.2% required re-enrolment. Similarly, 88.1% of eyes were recognized after pharmacologic dilation, with 11.9% needing re-enrolment. These results suggest that while specific ophthalmic interventions may temporarily affect recognition accuracy, iris biometrics largely remain reliable, with re-enrolment serving as an effective corrective measure.

Aslam TM et al. conducted a study to evaluate the impact of ocular diseases on iris recognition accuracy, analysing data from 54 patients with a range of eye conditions. They concluded that the iris recognition system exhibited strong resilience to most ocular conditions, including corneal edema, iridotomies, and conjunctivitis. However, the iris recognition system failed in some patients affected with acute inflammation of the iris (iritis/anterior uveitis), as there was an increase in Hamming distance in patients affected with uveitis compared to the control group [12].

Rathgeb and colleagues demonstrated that iris recognition systems remain resilient to minor anatomical and physiological changes. Our study supports their findings, further confirming that cataract surgery has minimal impact on iris recognition [13].

Ahmed et al. evaluated the long-term stability of iris recognition following ocular surgeries and reported a high accuracy retention, supporting the present study’s findings regarding uneventful surgeries [14].

Schuckers et al. highlighted the robustness of biometric systems to transient ocular changes such as mild inflammation or post-surgical recovery [15]. Our study also demonstrated that changes in pupil size and the natural healing process following surgery had no significant impact on the accuracy of iris recognition.

A single surgeon performed all the surgeries. Each patient in this study received a clear explanation of the image capture procedure and cooperated fully throughout the process. The exact mechanism by which the iris changes caused by the probe remains unclear. However, it is known that when the probe tip is directed toward the iris, it can cause tissue emulsification, leading to progressive atrophy of the iris. Even without direct contact, the energy released into the anterior chamber is believed to contribute to iris pigmentation.

The mean Hamming distance remained essentially unchanged following uneventful cataract surgery performed via phacoemulsification. The shift in iris patterns was minimal and did not significantly affect recognition accuracy for most individuals, who were still successfully identified.

Although postoperative factors such as temporary changes in pupil size or clarity of the ocular surface were considered, their impact on iris recognition was negligible.

This study did not yield any false matches; in other words, despite textural changes, no individual was incorrectly identified as someone else.

A key limitation of this study was its focus solely on uneventful cataract surgeries. Cases involving complications such as postoperative inflammation, corneal edema, or posterior capsular rent were excluded, thereby limiting the applicability of the findings to only ideal surgical outcomes.

Another limitation of the study was the short follow-up duration, which prevented the assessment of long-term effects such as lens opacification or other delayed complications. This study demonstrated that the majority of patients could be successfully identified after uneventful cataract surgery, with only a small number failing identification due to iris damage during the procedure. Therefore, we propose that iris biometric re-enrolment should not be mandated for all patients undergoing cataract surgery, as most individuals retain identifiable iris patterns postoperatively. Avoiding unnecessary re-enrolment would save time, reduce workforce demands, and prevent financial strain, especially since large-scale re-verification of identity through biometric systems would place a significant burden on government agencies.

## Conclusion

Iris recognition stands out as one of the most reliable methods of personal identification because each individual’s iris exhibits a uniquely intricate pattern that remains stable over time. Iris image matching remains unaffected in the majority of patients following uneventful cataract surgery. Therefore, iris recognition technology can be reliably used for biometric security purposes without requiring re-enrolment, as such surgeries do not significantly alter the iris pattern.
